# Political prioritization of prevention of mother-to-child transmission of HIV and syphilis: a qualitative comparative policy analysis in Ghana, Mozambique, and Sudan

**DOI:** 10.3389/fpubh.2026.1751253

**Published:** 2026-05-11

**Authors:** Sara Sulieman, Dadong Wu, Emmanuel Kwasi Afriyie, Munawar Harun Koray, Elsa Kanduma, Dong (Roman) Xu, Jing He

**Affiliations:** 1 School of Public Health, Southern Medical University, Guangzhou, China; 2 Department of Pharmacy Practice, Faculty of Pharmacy, University of Khartoum, Khartoum, Sudan; 3 Shenzhen Maternity and Child Healthcare Hospital, Women and Children’s Medical Center, Southern Medical University, Shenzhen, China; 4 Shenzhen Key Laboratory of Maternal and Child Health and Diseases, Shenzhen, China; 5 Komfo Anokye Teaching Hospital, Kumasi, Ashanti Region, Ghana; 6 Upper West Region Health Directorate, Wa, Upper West Region, Ghana; 7 Mozambique Health Committee, Maputo, Mozambique; 8 Mozambique Ministry of Health, Maputo, Mozambique; 9 Acacia Lab for Implementation Science, SMU Institute for Global Health (SIGHT) and Center for World Health Organization Studies, School of Health Management and Dermatology Hospital of Southern Medical University, Guangzhou, China; 10 Shenzhen Futian District Maternal and Child Health Hospital, Shenzhen, China

**Keywords:** Ghana, Mozambique, PMTCT, policy analysis, political prioritization, Sudan

## Abstract

**Background:**

Despite global commitments to eliminate mother-to-child transmission of HIV and syphilis, political and resource prioritization remains uneven across low- and middle-income countries. This has led to disparities in financial investment, policy implementation, and health outcomes. This study examines the factors shaping political prioritization of prevention of mother-to-child transmission (PMTCT) in Ghana, Mozambique, and Sudan using the Shiffman and Smith framework to understand how political, institutional, and contextual forces interact to influence national responses.

**Methods:**

A qualitative, cross-country comparative policy analysis was conducted based on document review from 3 data sources. Across countries, we included 21 government documents, 22 documents from non-governmental organizations, and 15 peer-reviewed articles selected through theoretical sampling. Both inductive and deductive thematic analyses were applied, with the latter guided by the Shiffman and Smith framework.

**Findings:**

Political prioritization of PMTCT was influenced by interrelated domains including actor power, ideas, political context, information systems, and financial resources. Ghana and Mozambique achieved higher prioritization through cohesive advocacy networks, effective issue framing, strong political commitment, reliable data, and sustained donor support. In contrast, Sudan’s limited progress reflected low political commitment due to fragmented leadership, weak coordination, inadequate data, and chronic resource constraints. The findings illustrate that progress depends not only on individual determinants but also on their interaction within national policy systems.

**Conclusion:**

Political prioritization of PMTCT results from the interaction of multiple interlinked factors rather than any single determinant. Strong advocacy, effective framing, reliable data, and sustained funding within supportive political environments foster commitment, as seen in Ghana and Mozambique. Coordinated advocacy, credible evidence, and predictable investment are essential to strengthen the PMTCT program by translating the global elimination goals into actionable national strategies.

## 1 Introduction

In 2014, the World Health Organization (WHO) launched the global initiative for the dual elimination of mother-to-child transmission (EMTCT) of HIV and syphilis (later expanded to include hepatitis B), setting the goal of eliminating both infections by 2030 as public health threats (1, 2). These include achieving at least 95% coverage for HIV and syphilis testing among pregnant women, as well as at least 95% treatment coverage for those who test positive (3). This initiative is closely aligned with Sustainable Development Goal 3, which calls for a reduction in maternal mortality, elimination of new pediatric HIV infections, and the broad control of infectious diseases, including AIDS (4–6). A range of global strategies and action plans have been launched to support national efforts toward EMTCT, emphasizing the delivery of comprehensive, integrated services for women living with HIV and syphilis (7, 8).

The dual EMTCT initiative presents a strategic opportunity to address two preventable infections simultaneously, thereby improving maternal and child health outcomes while strengthening the integration of services within existing health systems (9). Key to achieving EMTCT is the widespread implementation of prevention of mother-to-child transmission (PMTCT) interventions, which include HIV and syphilis screening during pregnancy, timely treatment (e.g., antiretroviral therapy (ART) and penicillin), and ongoing care throughout the maternal-child health continuum (2, 10). Evidence from low- and middle-income countries (LMICs) confirmed that such dual prevention strategies are feasible, acceptable, and cost-effective (11–13). The development and deployment of dual HIV/syphilis rapid diagnostic tests have further simplified and expanded testing coverage, enabling more efficient service delivery in both high- and low-HIV-burden settings (14, 15). Several countries—such as Cuba, Belarus, Oman, Thailand, Malaysia, and Armenia—have achieved WHO validation for dual EMTCT, demonstrating that the aspirational goal is attainable under conducive policy and programmatic environments (3, 16).

Nonetheless, many LMICs continue to face significant structural, political, and operational challenges in integrating PMTCT efforts into routine maternal and child health services (17). As a result, the global burden of maternally transmitted HIV and syphilis persists, particularly in regions with under-resourced health infrastructures. Globally, about 120,000 children acquire HIV annually from vertical transmission, mostly in sub-Saharan Africa (SSA). In 2023, 44% of new HIV cases were among women and girls, highlighting the ongoing feminization of the epidemic in the region (18). Without interventions like ART, maternal HIV transmission can reach 5–15% (19). Maternal syphilis is a major cause of adverse birth outcomes, including stillbirths, neonatal death, and congenital infection. In 2016, around 661,000 cases of congenital syphilis were reported worldwide, with the vast majority concentrated in sub-Saharan Africa (20, 21).

Ghana, Mozambique, and Sudan exemplify the ongoing challenges and divergent national responses to EMTCT in sub-Saharan African settings. Maternal HIV prevalence stands at 2% in Ghana and 7% in Mozambique (22, 23), while in Sudan it is considerably lower at 0.2% (24). Maternal syphilis prevalence remains comparatively high across all three countries, at 2.3% in Ghana, 2% in Mozambique (22, 23), and 1.4% in Sudan (24). Despite facing similar epidemiological threats, these countries differ markedly in their level of integration of PMTCT services and allocation of financial resources. As shown in Table 1, Ghana allocated $1.94 million to PMTCT in 2020—representing 44% of its national control budget of HIV and other sexually transmitted infections (STIs)—while Mozambique committed over $22.1 million in 2014 (25, 26). In contrast, Sudan allocated only $448,000, with PMTCT funding representing just 7% of its HIV prevention budget (27). These differences are reflected in key service coverage indicators. HIV testing coverage among pregnant women exceeds 88% in Ghana and 99% in Mozambique, compared to just 6% in Sudan. The ART coverage for PMTCT similarly reaches over 80% in Ghana and Mozambique, but only 4% in Sudan. Furthermore, while Ghana and Mozambique have made substantial progress in integrating syphilis testing and treatment into antenatal care, Sudan has only recently introduced syphilis screening into its national PMTCT policy, and implementation remains limited.

**Table 1:** Key PMTCT indicators in Ghana, Mozambique, and Sudan.

Indicator	Ghana	Mozambique	Sudan
Population size (in million)	33.8	33.6	50
Prevalence of maternal HIV	2	7	0.2%
Prevalence of maternal syphilis	2.3%	2%	1.4%
Annual PMTCT budget	$1,942,719.32	$22,100,000	$448,000
PMTCT share of HIV/STI control budget	44%	Not specified	7%
HIV testing coverage among pregnant women	88%	99%	6%
ART coverage for PMTCT	80%	97%	4%
Syphilis testing coverage among pregnant women	70%	78%	No data
Treatment coverage for maternal syphilis	112%	82%	No data

HIV, Human immunodeficiency virus; STI, sexually transmitted infection; ART, Antiretroviral therapy.

Source: Mozambique Ministry of Health (2022); Ghana AIDS Commission (2021); National AIDS Control Program, Ghana (2021); U.S. President’s Emergency Plan for AIDS Relief (2020); The World Bank Group (2014); The World Bank Group (2023), and Sudan National AIDS Control Program (2020).

These variations can be attributed to differences in the degree of political prioritization afforded to PMTCT in the three countries. As defined by Shiffman and Smith, political priority refers to the extent to which leaders consistently support a public health issue by mobilizing adequate financial, technical, and human resources (28). Importantly, the severity of the issue does not always drive political action. Instead, factors like cost-effectiveness, perceived feasibility, political visibility, and alignment with social norms often weigh more heavily in decision-making across both low- and high-income contexts (29, 30). Evidence from other LMICs, such as China, suggests that global norms, external funding, national policy networks, and stakeholder cohesion can significantly influence the salience of PMTCT on domestic agendas (31). In Ghana, Mozambique, and Sudan, research mostly focuses on clinical effectiveness, service-delivery outcomes, and coverage indicators (32–34). However, to date, no study has systematically investigated the political drivers—or barriers—underpinning the prioritization of PMTCT of HIV and syphilis in Ghana, Mozambique, or Sudan.

Given the persistent burden of maternal HIV and syphilis in these countries, there is an urgent need to understand how political dynamics shape national responses to this public health issue. This study applies a comparative policy analysis approach (35, 36), informed by Shiffman and Smith’s framework on the determinants of political priority in global health to examine the institutional, contextual, and stakeholders-related factors that affect political attention to PMTCT (28). By identifying key enablers and constraints, the research aims to generate actionable insights that can support countries in aligning their national strategies with the global EMTCT targets for 2030. Beyond the case countries, the findings offer broader implications for strengthening the prioritization of maternal and child health in LMICs facing similar health and governance challenges.

## 2 Methods

### 2.1 Study design

This study employed a qualitative, cross-country comparative policy analysis based on the review of policy-relevant documents to assess the prioritization of PMTCT for HIV and syphilis on the national agendas of Ghana, Mozambique, and Sudan. A case study approach was adopted to enable an in-depth examination of complex health policy processes within real-world contexts, allowing exploration of causal pathways and mechanisms underlying policy prioritization (35, 37, 38). The comparative design was particularly valuable for identifying how contextual factors influence policy adoption and implementation, and for highlighting variations that support theory-driven conclusions, even if findings are not universally generalizable (39, 40). In this study, findings are not intended to be widely generalizable; however, they offer transferable insights for other LMIC contexts with similar political and resource constraints. The reporting of this study adheres to the Standard for Reporting Qualitative Research (SRQR) (41).

### 2.2 Policy framework

This study is guided by the Shiffman and Smith Framework, a well-established model for analyzing the determinants of political priority for global health initiatives (28). Recognized as one of the most comprehensive health policy analysis frameworks (42), it examines why certain health issues fail to gain the level of political support they warrant. The framework has been widely applied to studies on maternal and infant health, mental health, and reproductive health policies at both global and national levels (43–46). It is also widely used in LMICs and SSA (47, 48). As shown in Table 2, Shiffman and Smith identified 11 factors influencing political prioritization, organized into four key domains: the power of actors involved in advocating for the issue; the influence of ideas shaping its framing and explanation; the political context affecting prioritization; and the characteristics of the issue that determine its perceived urgency.

**Table 2:** The Shiffman and Smith framework for political prioritization.

Domain	Description	Determinants of political priority
Actor power	The capabilities of the individuals and organizations involved in the issue.	1. Policy community cohesion 2. Leadership 3. Guiding institutions 4. Civil society mobilization
Idea	The perspectives of those involved with the issue and how they interpret and represent it.	5. Internal frames 6. External frames
Political contexts	The environments in which actors function.	7. Policy windows 8. Global governance structure
Issue characteristics	Features of the issue.	9. Credible indicators 10. Severity 11. Effective interventions

### 2.3 Study settings

Ghana, Mozambique, and Sudan are LMICs in SSA (49). These countries share high donor dependence for STI and PMTCT programs with marked differences in political prioritization of PMTCT, making them suitable cases for comparative analysis. Additionally, these countries differ in sublocation and regional affiliations, with Ghana and Mozambique falling under the African region and located to the West and South, while Sudan located to the northeast of Africa, belongs to the Eastern Mediterranean region (50). This may have an impact on health governance at the regional and national level, and collaborative action to tackle health issues (51).

This comparative study sheds light on why Ghana and Mozambique have allocated moderately high political and financial resources to PMTCT, in contrast to Sudan, which has not. Examining these cases together enables identification of the factors influencing policy prioritization in these contexts, which can guide strategies to strengthen PMTCT initiatives in similar settings. The choice of these countries was also strategic, utilizing existing relationships with policymakers to gain access to crucial policy documents and data sources.

### 2.4 Researchers’ positionality

The six-member research team included scholars from Ghana, Mozambique, Sudan, and China, combining insider perspectives with contextual knowledge and networks, and outsider perspectives offering comparative insight and analytical distance. Team members were affiliated with universities, a tertiary hospital, national and regional health departments, and a non-governmental organization (NGO), with expertise spanning global health, health policy, maternal and child health, hospital management, and implementation science. The composition of two senior researchers and three doctoral students provided both extensive experience and emerging research perspectives. Potential influence of prior professional roles and policy engagement on interpretation was addressed through regular reflexive discussions, enhancing the credibility and trustworthiness of the comparative policy analysis.

### 2.5 Sampling and data collection

This study relied on secondary data sources to assess the political prioritization of PMTCT, including:

1. Published literature.
2. Governmental documents (e.g., technical guidelines, epidemiological reports, implementation plans, meeting and training materials).
3. Reports from international agencies (e.g., WHO, Joint United Nations Program on HIV/AIDS (UNAIDS), United Nations International Children’s Emergency Fund (UNICEF), President’s Emergency Plan for AIDS Relief (PEPFAR), The World Bank, and relevant NGOs).

Inclusion criteria for documents:

- Languages: English, Portuguese, or Arabic (the official languages of Ghana, Mozambique, and Sudan, respectively)
- Publication years: 1990–2023
- Content: Qualitative or quantitative data on factors influencing the prioritization of PMTCT of HIV and syphilis in the three countries, aligned with the Shiffman and Smith framework

Data collection occurred in multiple rounds from February 2022 to December 2023. Across the three countries, 21 government documents, 22 non-governmental organization documents, and 15 research articles were included, using theoretical sampling and focusing on documents with data related to the political prioritization of PMTCT in the study settings. Documents were weighted equally, with analytical importance determined by thematic convergence rather than document types. Data collection, coding, and analysis were conducted iteratively to achieve thematic saturation within each theme (i.e., factor affecting priority) for each country. Saturation was reached when no additional data were found with further document collection, and each country case was sufficiently understood. SS, EKA, and EK gathered government documents. However, except for two policy documents from Sudan (24, 52), all data used to support the conclusions of this article are drawn from publicly available reports. Organizational reports and relevant literature were sourced from public databases, websites, and search engines. Supplementary material 1 includes a list of documents used to support the results of this study.

### 2.6 Data analysis

Data was analyzed using thematic analysis, employing both deductive and inductive approaches:

- Deductive coding applied Shiffman and Smith’s 11 political prioritization factors, including policy community cohesion, leadership, guiding institutions, civil society mobilization, framing (internal/external), policy windows, global governance structure, credible indicators, severity, and intervention effectiveness.
- Inductive coding allowed for flexibility, enabling the identification of additional factors outside the framework. Identification of themes was evident by their recurrence across multiple documents and country cases and by their strong relevance to policy prioritization.

Data from different sources (governmental, non-governmental, and academic literature) were synthesized and categorized under the same themes when referring to similar policy concepts. Triangulation was used whenever possible to reduce bias and enhance the trustworthiness and credibility in the data analysis process. To ensure rigor, an iterative data collection and analysis process continued until thematic saturation was reached, thereby supporting the identification of the emergent theme. NVivo 12 (QSR International) was used to facilitate data management and coding. Coding was initially completed by SS and EKA, and double-checked by DW, an expert in policy analysis and qualitative research. Any discrepancies were resolved by discussion. We examined how each factor affected policy and resource attention, and how these translated into service delivery across contexts. Our conclusion regarding the determinants of prioritization is informed by in-depth triangulation across multiple data sources, cross-case comparison, and alignment with the Shiffman and Smith framework.

## 3 Results

The findings showed how PMTCT initiatives for HIV and syphilis align with the four key domains of the Shiffman and Smith framework: actor power, the strength of the idea, political context, and issue characteristics. Additionally, resource provision emerged inductively as an important factor in prioritizing PMTCT in these settings. Table 3 summarizes the main findings.

**Table 3:** Summary of key factors identified.

Factors	Ghana	Mozambique	Sudan
1. Policy community cohesion 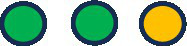	Strong collaboration among national and international actors within the PMTCT working groups.	Multi-agency and cross-country collaborations indicate a cohesive policy network.	Actor involvement is largely limited to guidelines and strategies, with minimal practical implementation.
2. Leadership 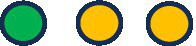	First Lady’s OAFLAD advocacy during its 15th and 16th sessions elevated PMTCT guidelines on the national agenda.	First Lady’s OAFLAD role at the 2012 Summit of Heads of State helped expand PMTCT service coverage nationally.	First Lady’s OAFLAD involvement offered minimal support, with no tangible service expansion or coverage gains.
3. Guiding institutions 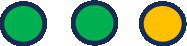	Overlapping responsibilities between NACP and the Family Health Division create role ambiguity; decentralization to lower health system levels promotes PMTCT.	Decentralization attracted donor support and funding for PMTCT.	Weak coordination and management among guiding institutions contributed to a fragile health system and poor preparedness.
4. Civil society mobilization 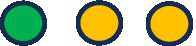	Christian Health Association of Ghana and Rural Watch piloted the Mentor Mother initiative, attracting national and international support.	Grassroots organizations implement strategies but have limited engagement in policy processes.	Civil society organizations and volunteers did not participate in advocacy for PMTCT.
5. Internal frames 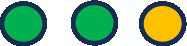	PMTCT framed as vital for saving children’s lives and reducing HIV infections; maternal deaths framed as societal issues, prompting programs such as LEAP.	Broad consensus on PMTCT’s role in HIV control, supported by the Child Health Card program, which improved newborn HIV testing.	Perspectives on HIV interventions are mixed, limiting funding and resource allocation.
6. External frames 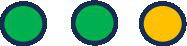	PMTCT framed as a health right, aligning with UNGASS declarations and women’s/children’s rights commitments.	Right-to-health framing, especially for vulnerable groups, motivates donor funding (DREAM, PEPFAR).	Lacks compelling external framing to mobilize support for PMTCT.
7. Policy windows 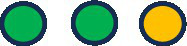	Global and regional commitments (UNGASS, MDGs, SDGs) emphasize prioritizing PMTCT of HIV and syphilis.		UNGASS and MDGs prompted PMTCT’s inclusion in the national agenda, but resources remain insufficient.
8. Global governance structure 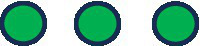	Dual EMTCT and TAP multinational initiatives created a favorable environment for PMTCT policy enactment and implementation.		Weak governance structure has failed to elevate PMTCT on the policy agenda.
9. Credible indicators 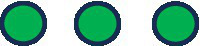	Robust HIS with standardized PMTCT monitoring supports data-driven decision-making.		Fragmented HIS limits the availability of reliable HIV/syphilis indicators.
10. Severity 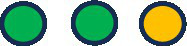	Globally, 2% of children living with HIV are in Ghana; maternal syphilis prevalence 2.3%.	10% of HIV-positive children globally are in Mozambique; maternal syphilis prevalence ~2%.	Low maternal HIV prevalence (0.2%) and conflicting views on the severity of maternal syphilis (1.4–9%) reduce PMTCT priority.
11. Effective intervention 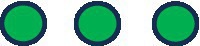	Multiple studies and pilots confirm effectiveness of PMTCT interventions for prevention of vertical transmission of both infections.		PMTCT for HIV shown cost-effective only since 2020; no evidence available for syphilis.
12. Resource provision 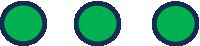	Strong technical/financial support from CDC, PEPFAR, Global Fund, WHO, and other UN agencies.	PEPFAR, WHO, and Global Fund provide substantial support for PMTCT.	International aid restrictions have hindered PMTCT progress.

The three circles depict the strength of evidence on each factor’s impact on PMTCT’s political priorities. Green indicates highly influential factors, whereas orange signifies factors with lesser impact.

CDC, Centers for Disease Control and Prevention; DREAM, Drug Resource Enhancement against AIDS and Malnutrition; HIS, Health Information System; LEAP, Livelihood Empowerment Against Poverty; MDGs, Millennium Development Goals; MTCT, Mother-to-Child Transmission; NACP, National AIDS Control Program; OAFLAD, Organization of African First Ladies for Development; PEPFAR, President’s Emergency Plan for AIDS Relief; PMTCT, Prevention of Mother-to-Child Transmission; SDGs, Sustainable Development Goals; TAP, Treatment Acceleration Program; UNGASS, United Nations General Assembly Special Session; WHO, World Health Organization.

### 3.1 Actor power

#### 3.1.1 Cohesion of the policy community

Policy communities that achieve consensus on key issues are more likely to gain political support, as unified positions enhance credibility among decision-makers (53). In Ghana, Mozambique, and Sudan, PMTCT policymaking is largely multi-sectoral, involving public and private sectors, civil society, and international agencies (54, 55). Periodic multi-stakeholder meetings provide platforms for consensus-building and coordination (56–58).

In Ghana and Mozambique, advocacy networks have strengthened policy cohesion, increasing consensus and resource allocation. For example, Ghana’s collaboration with WHO and UNICEF in 2019 integrated early infant diagnosis and prophylaxis as essential PMTCT components (58). Mozambique’s partnerships with the OPEC Fund, the WHO African Region, and the WHO/Italian Initiative in 2001 enhanced health system capacity and scaled up community-based PMTCT efforts (59). In Sudan, while multiple actors reached consensus at the policy development level (52), this has not translated into a strong political influence to drive implementation. Additionally, private sector involvement remains minimal (60).

#### 3.1.2 Leadership

The presence of strong advocates is vital to champion a response at all levels and influence the policy environment for the benefit of the issues (61). The Ordinary Assembly of the African First Ladies against HIV/AIDS (known currently as Organization of African First Ladies for Development, OAFLAD), which provides a platform for first ladies to champion PMTCT initiatives as part of broader efforts to combat HIV/AIDS (62). PMTCT has gained support from the first ladies in the three countries; however, it has a different impact on policy and service provision. At the 2012 Southern African Development Community Summit, Mozambique’s First Lady, Dr. *Maria Dia Luz Guebuza*, presented the Declaration for the EMTCT of HIV, endorsed by regional leaders. As a patron of the Global Plan Toward the Elimination of New HIV Infections among Children by 2015 and Keeping Their Mothers Alive, Dr. *Guebuza* mobilized national and regional efforts to achieve zero new HIV infections in children (63). Similarly, in Ghana, First Lady *Lordina Mahama* advocated for the elimination of new HIV infections among children during her tenure as President of OAFLAD at the 15th Ordinary Assembly (64, 65). In Sudan, after participating in the OAFLAD meeting in Ethiopia in 2011, the First Lady launched campaigns to promote PMTCT efforts, which led to expanding the number of PMTCT service sites from 27 in 2009 to 70 in 2011 (56). However, this did not result in a tangible increase in service coverage. Leadership in all three countries remains largely confined to top-level figures; engagement from health policymakers, healthcare providers, and academic institutions was not observed. Importantly, these leadership initiatives do not encompass PMTCT of syphilis.

#### 3.1.3 Guiding institution

Guiding institutions and their coordinating mechanisms have a significant influence on the initiative’s survival (66). In Ghana, Mozambique, and Sudan, the National AIDS Program and Women & Child Health Departments serve as the primary governmental bodies overseeing PMTCT services (23, 52, 55).

However, fragmented leadership structures in Ghana and Sudan have hindered effective coordination among key stakeholders. This in Sudan influenced human resource management and the integration of HIV care into antenatal services, impacting various departments and facilities, including reproductive health programs, HIV services, and laboratory networks (60). In Ghana, weak communication channels between different levels of the health system have impeded the effective dissemination of PMTCT services, limiting accessibility and uptake (23, 67).

However, decentralization as a mechanism for PMTCT services management has greatly enhanced PMTCT service delivery in Ghana and Mozambique. In Ghana, this approach increased the number of PMTCT centers from 135 in 2005 to 793 by 2009, facilitating the introduction of syphilis PMTCT services (54, 67). Decentralization in Mozambique attracted numerous organizations to provide financial and technical support, including PEPFAR, United States Agency for International Development (USAID), UNICEF, the World Bank, the Bill Clinton Foundation, the Bill & Melinda Gates Foundation, Columbia University, and the Corporation for National and Community Service. This multi-stakeholder engagement led to the expansion of PMTCT services and improved program reach and effectiveness (59).

#### 3.1.4 Civil society mobilization

Grassroots organizations are vital for advocacy efforts, ensuring accountability for policy changes at the national and international levels (68). Ghana’s organizations attract political leaders from local and international agencies. In Mozambique and Sudan, although civil society plays a key role in PMTCT, it primarily focuses on implementation rather than policy advocacy.

In Ghana, civil society’s impact is evident through the efforts of the Christian Health Association of Ghana and Rural Watch, which helped elevate PMTCT as a national priority by implementing the Mentor Mothers initiative. The Mentor Mothers initiative—where HIV-positive mothers who have undergone the PMTCT process in antenatal care (ANC) provide peer support to other HIV-positive pregnant and lactating women—was formally adopted by the government. This initiative gained additional support from international agencies such as Mother2Mothers, strengthening its reach and impact on PMTCT service delivery (23, 58, 69).

The Mentor Mothers program was also expanded to Mozambique through collaborative efforts led by WHO and PEPFAR, in partnership with the Mozambican Ministry of Health (MOH) and USAID, under the Partnership for HIV-Free Survival initiative (70, 71). By 2018, Mother2Mothers joined these efforts, further strengthening support networks for women in PMTCT programs (72). However, the scope of these efforts remains restricted to implementing policies rather than advocating for policy changes. Similarly, in Sudan, national NGOs are increasingly engaged in HIV education, ART and PMTCT services, aligning with government objectives for broader community participation (73). However, these organizations face significant barriers to influencing policy, including limited technical capacity, weak institutional recognition, and restricted access to decision-making platforms. As a result, their impact on PMTCT policy formulation remains limited (60).

### 3.2 Ideas

#### 3.2.1 Internal frame

The frame alignment has a potential impact on social and resource mobilization (74). The strong consensus among national and international leaders and scientists regarding PMTCT guidelines has established PMTCT as a key national priority in Ghana and Mozambique (55, 75). In Ghana, the government has strengthened its commitment to maternal and child health services, emphasizing the reduction of new HIV infections among children as a vital public health and development priority (75). Maternal deaths from HIV and the increase in orphans due to AIDS are seen as a societal issue threatening family integrity. Consequently, PMTCT is included in Ghana’s Social Welfare agenda by the Ministry of Manpower and Employment, establishing the Livelihood Empowerment Against Poverty program. This program has enhanced access to social services for pregnant women and children and reduced mother-to-child transmission (MTCT) rates (54). The Child Health Card initiative in Mozambique, launched on 1 June 2009, as part of a broader child survival strategy, significantly increased newborn HIV testing—a key PMTCT intervention. Testing rates rose from 14% in 2008 to 30.9% by the end of 2009, reflecting national investment in PMTCT strategies (55, 57). In Sudan, perspectives on effective HIV interventions are less unified. The World Bank has advocated for targeting at-risk populations over community-wide interventions like PMTCT, influencing national priorities (27). While many stakeholders accept this approach, government support for PMTCT remains visible, as reflected in the nationwide expansion of PMTCT centers.

#### 3.2.2 External frame

The framing of health issues significantly affects their presence on national and global policy agendas, especially in appeals to political leaders who manage resources (76). A strategic and compelling frame, such as a human rights and social justice perspective, can elevate an issue’s prominence, driving policy action and funding commitments (77). PMTCT has been successfully framed as a human rights issue in Ghana and Mozambique, reinforcing governmental and international commitments. Since 2001, Ghana has committed to the rights of people living with HIV by signing the United Nations General Assembly Special Sessions (UNGASS) declarations. The government has enacted policies to empower women with HIV, ensuring access to prevention, treatment, and care. These initiatives align with the Convention on the Rights of the Child and Ghana’s goal of eradicating gender-based discrimination in healthcare services (78, 79). Mozambique has utilized the right-to-health framework to gain political support for PMTCT. A key example is the Drug Resource Enhancement against AIDS and Malnutrition Program (DREAM), funded by PEPFAR, which provides ART to pregnant and breastfeeding women as part of PMTCT services. Since launching in 2002, the DREAM program has focused on improving maternal HIV care, and by 2022, it accounted for 8% of PEPFAR Mozambique’s overall budget, underscoring its role in a rights-based PMTCT approach (80, 81). Unlike Ghana and Mozambique, where human rights narratives have helped secure policy attention and donor investments, Sudan lacks a cohesive advocacy strategy, reducing its visibility on global health platforms. This weak external positioning has contributed to lower resource mobilization and limited policy influence.

### 3.3 Political context

#### 3.3.1 Policy window

A policy window arises when various conditions create openings for policy change and enable policy groups to impact decision-making (82). Key international frameworks such as UNGASS, the Millennium Development Goals (MDGs), and the Sustainable Development Goals (SDGs) have been pivotal in promoting PMTCT initiatives for HIV and syphilis in Ghana, Mozambique, and Sudan (57, 79, 83). The global commitment to the MDGs significantly contributed to positioning the MTCT of syphilis as a major public health concern. The goals—MDG 4 (reducing child mortality), MDG 5 (improving maternal health), and MDG 6 (combating HIV/AIDS, malaria, and other diseases)—aligned closely with the EMTCT of syphilis, prompting WHO investments in the EMTCT programs of Ghana and Mozambique (12, 78). The WHO African Region Office reports that Ghana and Mozambique have pledged to several global and regional agreements focused on maternal and child survival and HIV prevention, thereby intensifying political pressure to dedicate resources to integrate PMTCT (78). Furthermore, both nations were recognized as part of the 22 priority countries in the Global Plan to eliminate new HIV infections among children by 2015, highlighting global support for the expansion of PMTCT programs (84). Sudan, a signatory of the Millennium Summit’s pledge alongside 189 nations to lower maternal and child mortality and to prevent HIV infections, has integrated these commitments into its strategic STIs control initiatives (85). Nonetheless, the integration of PMTCT is still lacking, requiring additional efforts to fully implement and maintain EMTCT programs.

#### 3.3.2 Global governance structure

The global governance structure encompasses international norms, legislation, and treaties, providing a collaborative foundation for health initiatives. The WHO with advocacy from other UN agencies, launched several strategies and global and regional action plans that support the PMTCT, which included the 2014 and 2017 *Global Guidance on* Criteria and Processes for Validation for Dual EMTCT of HIV and syphilis (1, 3), as well as commitments to the prevention of new HIV infections among children and keeping their mothers alive (6). In line with these efforts, the WHO African and East Mediterranean Regional Offices have created platforms for member countries to engage on shared PMTCT strategies, integration, and implementation efforts (78, 86). Ghana, Mozambique, and Sudan have synchronized their national strategies with global and regional objectives.

Nonetheless, the global governance framework seems to place a greater emphasis on PMTCT initiatives in Ghana and Mozambique than in Sudan, evident in the degree of PMTCT integration and execution within each nation’s health policies. The Dual EMTCT Plan has identified Ghana and Mozambique as priority countries, granting them increased support in data collection, monitoring, and decision-making to advance PMTCT of HIV and syphilis. The Treatment Acceleration Project (TAP) unites nations and agencies (WHO, World Bank, and United Nations Economic Commission for Africa) in their PMTCT objectives. It provides a collaborative framework that strengthens efforts in Ghana and Mozambique, reinforcing the PMTCT initiative and fostering cohesive action toward EMTCT goals (87).

### 3.4 Issue characteristics

#### 3.4.1 Credible indicators

The availability of robust and specific health indicators has the potential to advocate for health issues (88, 89). Ghana and Mozambique have strong health information systems (HIS) that support PMTCT decision-making. They established a robust monitoring system for PMTCT of HIV, ensuring reliable indicators for strategic planning and policy formulation. The national HIS of PMTCT in Mozambique is simple, timely, flexible and represents all ANC clients in the country (90). The Mozambican government routinely analyzes service delivery, including HIV and syphilis testing and treatment, using the WHO estimation tool for PMTCT trends (91, 92). Ghana employs the Estimation and Projection Package to develop national and sub-national HIV trends, providing precise, granular data for guiding geographic planning and monitoring (58). The same applies to the data collection on PMTCT of syphilis; in Ghana, the monitoring system allows for accurate tracking, which enhances informed decision-making (67). The WHO has recognized Ghana and Mozambique as two of the 12 countries selected for globally eliminating congenital syphilis, owing to their dependable PMTCT indicators. Only countries with strong monitoring systems qualify for WHO investment in this area (12).

Conversely, Sudan’s PMTCT HIS is significantly lagging. The 2022 WHO Assessment of HIS in Sudan highlights outdated and incomplete reports. The HIS excludes many primary healthcare facilities and most private providers (93). The 2019-ANC Sentinel Sero Survey was conducted at only 37 sites (24), raising concerns about representativeness. Furthermore, key indicators, such as mother–child pairing outcomes, are untracked due to the lack of Polymerase chain reaction testing for infants of HIV-positive mothers (56). A shortage of syphilis indicators also obstructs effective monitoring of MTCT. The lack of information and unreliability regarding MTCT in Sudan are essential factors that lead to low coverage of services in the country (94). Importantly, the scarcity of PMTCT indicators in Sudan reflects not only technical deficiencies in data systems but also historically low political emphasis on PMTCT, coupled with the recent inclusion of PMTCT for syphilis in the national health agenda, which has led to limited policy documentation and limited routine data availability.

#### 3.4.2 Severity

Severity is an essential criterion for priority setting (95). The high severity of maternal HIV and syphilis draws considerable attention to MTCT in Ghana and Mozambique, in contrast to Sudan, where the high incidence of maternal syphilis fails to capture policymakers’ interest, primarily due to conflicting evidence. Ghana and Mozambique are two of the 16 countries with 90% of pregnant women living with HIV, and are among the 23 most responsible for rising new HIV infections among children. Mozambique accounts for 10% of this increase, while Ghana accounts for 2% (96). In line with this, Ghana adopted new WHO guidelines for HIV prevention and treatment as an expedited plan toward EMTCT of HIV (97). Likewise, in response to the troubling HIV burden among children in Mozambique, particularly evident in the 2010–2014 third strategic plan, PEN III, and the consequent PEN IV, the government recognized PMTCT as one of the three primary objectives for HIV control efforts (98–100). In Sudan, contradictions regarding the severity of MTCT of HIV and syphilis significantly contribute to its low priority. The strategic plan for 2010–2014 highlights PMTCT of HIV as a critical prevention approach in light of the concerning MTCT rates presented by the 2009 National Projection Estimates. These estimates indicated that 29% of children born to HIV-positive mothers could be infected, with this figure expected to increase to 35.74% by 2011, underscoring the pressing necessity for PMTCT interventions (56, 83). Recently, maternal HIV prevalence has been debated; sentinel sero-surveillance in 2019 indicated 0.2% (24), while a 2018 meta-analysis estimated it at 1.4% (101). Some studies reported maternal syphilis prevalence as high as 3–9% (102, 103). Although reported by national data as only 1.4% (24).

In 2009, the Ghana Health Service implemented routine syphilis screening for ANC attendees across the country, responding to an increase maternal syphilis from 0.4% in 2003 to 6.5% in 2008 (67, 104). Since the 1990s, research conducted in Mozambique has revealed significant syphilis rates among attendees of ANC, supporting the need for a community-based approach that includes regular screening, health assessments, and treatment (105). The MOH managed to include syphilis screening in the national health agenda before 2000 (106). Moreover, the international community has offered significant support in Ghana and Mozambique, countries that have some of the highest rates of prenatal syphilis, resulting in the WHO investment in PMTCT initiatives (12).

#### 3.4.3 Effective intervention

Cost-effectiveness guides the prioritization of interventions by pinpointing those that offer the most significant health benefits for the investment, to optimize overall health (107, 108). A primary outcome of TAP in Ghana and Mozambique is demonstrating the cost-effectiveness of the PMTCT program and enhancing service delivery efficiency (87). In Ghana, the ART option B+, recommended for PMTCT interventions, will prevent HIV in 668 infants born to HIV-positive mothers. This option is the most cost-effective method for the PMTCT of HIV in resource-limited settings, including multiple pregnancies, like those in Ghana (109). According to the AIDS Investment Framework 2013; Ghana National Plan 2011–2016; and the Fast-track targets of 95–95–95 by 2025, the PMTCT program was identified as a key strategy for an effective HIV response. This led to increased attention and priority for PMTCT in strategic plans from 2016 to 2025 (97, 110).

Sudan adopted PMTCT of HIV in 2007; however, in 2012, a study suggested that implementing PMTCT interventions is a cost-effective strategy only in areas with high HIV incidence (94). Given Sudan’s low HIV epidemic, a cost-effectiveness study suggested focusing resource allocation for HIV control on specific regions and at-risk populations to prevent 29,300 additional HIV-related DALYs (23%), indicating that nationwide initiatives like PMTCT services were effective only after 2020 (27).

Regarding PMTCT of syphilis, the WHO recommends the dual HIV/Syphilis intervention to improve the acceptance and uptake of antenatal syphilis testing and treatment (1). Consequently, Ghana and Mozambique introduced dual EMTCT for HIV and syphilis to improve maternal testing. Both countries use rapid point-of-care tests for screening and Benzathine penicillin for treatment. These interventions are safe, sensitive, and specific, yielding reliable results. They are simple to use, resource-efficient, and readily available, making them suitable for resource-limited settings (104, 111).

### 3.5 Resource provision

Financial support emerged, beyond the initial framework, as a critical factor shaping the prioritization of PMTCT. Availability of funds ensures sufficient resources for both implementation and sustainability (112). Mozambique and Ghana have benefited from substantial donor funding, while Sudan has struggled with limited financial allocation for PMTCT. As a priority for PMTCT funding, Mozambique has received significant support from PEPFAR and the Global Fund, the most prominent donors for global PMTCT programs. In 2014, it allocated $22.1 million to PMTCT services, with PEPFAR contributing 75% and the Global Fund 10% (26). PEPFAR directly supports Mozambique’s MOH through government-to-government funding for PMTCT programs and through AJUDA sites (Analyzing Joint Underperformance and Determining Assistance), medical centers offering comprehensive HIV services covering 636 of 1,055 sustainability sites (80). In 2022, PEPFAR identified 187 pediatric and PMTCT sites for intensive technical assistance, further enhancing Mozambique’s PMTCT capacity (80). In Ghana, PMTCT expenditure comprises 44% of the National AIDS Control Program budget. Funds mainly come from donors, including the Global Fund, USAID (PEPFAR and the Centers for Disease Control and Prevention), the Joint United Nations Team on AIDS (UNICEF, WHO, UNAIDS), and the West African Health Organization (25). The TAP in Ghana and Mozambique enhanced HIV PMTCT response in the early stages with financial, technical support, and global collaborations (87, 113).

Similarly, PMTCT of syphilis in Ghana and Mozambique has received significant resources. In 2012, the WHO launched an initiative for the global elimination of congenital syphilis in 12 countries with the highest maternal syphilis burden. Approximately $17.4 million was allocated over 4 years. Ghana and Mozambique benefit from these funds in capacity building, monitoring, and evaluating the implementation of the PMTCT services (12).

In Sudan, the 2013 National AIDS Spending Assessment reported programmatic HIV spending of 6.4 million USD, with only 7% for PMTCT services. This limited funding excluded PMTCT from cost-effective HIV policy alternatives (27).

## 4 Discussion

For an issue to rise to the government’s agenda and gain high priority, it must capture sustained political attention—a benchmark that many important health concerns fail to meet and one that remains poorly understood (114). This study, drawing on cases from Ghana, Mozambique, and Sudan, provides a detailed examination of the determinants of political prioritization of PMTCT of HIV and syphilis in LMICs. The WHO has created policy documents, guidelines, and frameworks defining global and national targets to eliminate mother-to-child HIV and syphilis transmission. Countries were provided with technical assistance, capacity-building tools, and endorsement mechanisms to support the adoption, implementation, and monitoring of PMTCT/EMTCT programs. However, country responses varied. Ghana and Mozambique demonstrated substantially greater progress by adopting and implementing the WHO dual elimination plan and achieving tangible outcomes, whereas Sudan, despite a formal commitment, made limited progress (Table 1). This study highlights how PMTCT response is shaped by interactions among political, institutional, and contextual factors and underscores the importance of translating global EMTCT goals into actionable public health policies. The analysis reveals that strong actor networks, effective framing strategies, alignment with global health commitments, reliable data systems, and adequate financial investment were key drivers of higher political prioritization in Ghana and Mozambique. In contrast, Sudan’s limited progress reflected fragmented leadership, weak policy frameworks, an unfavorable policy environment, inadequate health information systems, and chronic funding shortages.

### 4.1 Key factors shaping PMTCT prioritization

Actor power and institutional coordination: The findings suggest that political priority of PMTCT is shaped by the presence of relevant actors, the strength of coordination and leadership within policy communities. In Ghana and Mozambique, strong policy communities and leadership were crucial to advancing PMTCT resulting in effective policy formulation and implementation. In Sudan, although the policy community reached consensus on developing PMTCT policies, poor coordination among actors hindered implementation, leading to limited resource allocation and service coverage. Similar patterns have been observed in other African countries. For example, in Uganda, cohesive and diverse policy communities have played a critical role in prioritizing maternal and child health issues on national agendas (115), while in Ethiopia, inadequate multisectoral engagement has been identified as a barrier to sexual and reproductive health services for adolescents (116).

The participation of First Ladies in senior advocacy roles across the three countries provided significant support for PMTCT. This aligns with earlier evidence emphasizing the importance of leadership in policy change for PMTCT integration (84). However, a shortage of lower-level advocates can weaken administration and compromise service delivery, hampering further progress (16). In Sudan and Mozambique, civil society participation remains limited, focused primarily on service delivery rather than policy-making. Limited advocacy capacity, inadequate funding, donor competition, and restrictive political environments further constrain civil society engagement.

Framing and ideas: Framing maternal health issues within the context of human rights and social justice enables them to receive greater prioritization on the global stage (77). This aligns with Ghana and Mozambique, where PMTCT was positioned as both a human right and a maternal and child health issue, facilitating alignment with the global agenda. In contrast to Sudan, where such framing is absent, consequently, has failed to attract adequate political focus.

Policy context: As with many public health challenges in LMICs, the MDGs and SDGs provided critical political opportunities to position PMTCT as a national priority, helping sustain political commitments and mobilize resources for elimination goals (88, 117). In Ghana and Mozambique, the MDGs, SDGs, and the Global Plan for Eliminating New HIV Infections among Children were leveraged to integrate PMTCT into national health strategies, thereby securing both domestic and international investment. Other LMIC studies also identify political environment and critical events as significant influences on HIV policy-making (118, 119). Sudan, however, missed key opportunities to capitalize on global health commitments. Although a signatory to major international agreements, it has struggled to translate commitments into effective policy action. Moving forward, Sudan should seize emerging global policy windows—such as the SDGs and Universal Health Coverage frameworks—to integrate PMTCT within broader national health strategies.

Issue characteristics and credible indicators: Differences in HIS capacity and the availability of credible PMTCT data help explain variation in political priority across the three countries. Reliable indicators of maternal HIV and syphilis burden in Ghana and Mozambique, together with strong evidence on PMTCT effectiveness, have attracted international attention and policy support. Context-specific, evidence-informed policy choices play a pivotal role in shaping PMTCT priorities and advancing broader maternal and child health initiatives (31, 120, 121). By contrast, inadequate PMTCT data collection in Sudan has restricted visibility, policy responsiveness, and donor investment. Prior studies have also shown that Sudan’s insufficient monitoring systems undermine planning for other key health issues (122). Strengthening national surveillance, routine data reporting, and HIS integration is therefore critical for improving policy prioritization and monitoring progress (123).

Resource provision: Financial resources were found to play a decisive role in determining PMTCT prioritization and sustainability. Ghana and Mozambique benefited from substantial donor funding, particularly from PEPFAR and the Global Fund, as well as multinational initiatives such as TAP and WHO’s investment in maternal syphilis programs. Prior research consistently identifies financial and technical assistance from international partners as key to advancing maternal and child health, including MTCT prevention (115, 124–127). Conversely, fragmentation in advocacy efforts and the absence of effective health services are largely attributed to resource constraints (128, 129). In Sudan, the scarcity of both domestic and external funding constrained program expansion, training, and community outreach, resulting in persistently low PMTCT coverage. To address these gaps, Sudan must increase domestic investment in PMTCT and explore opportunities for public–private partnerships.

### 4.2 Interacting factors influencing the political priority of PMTCT

**Figure 1:**
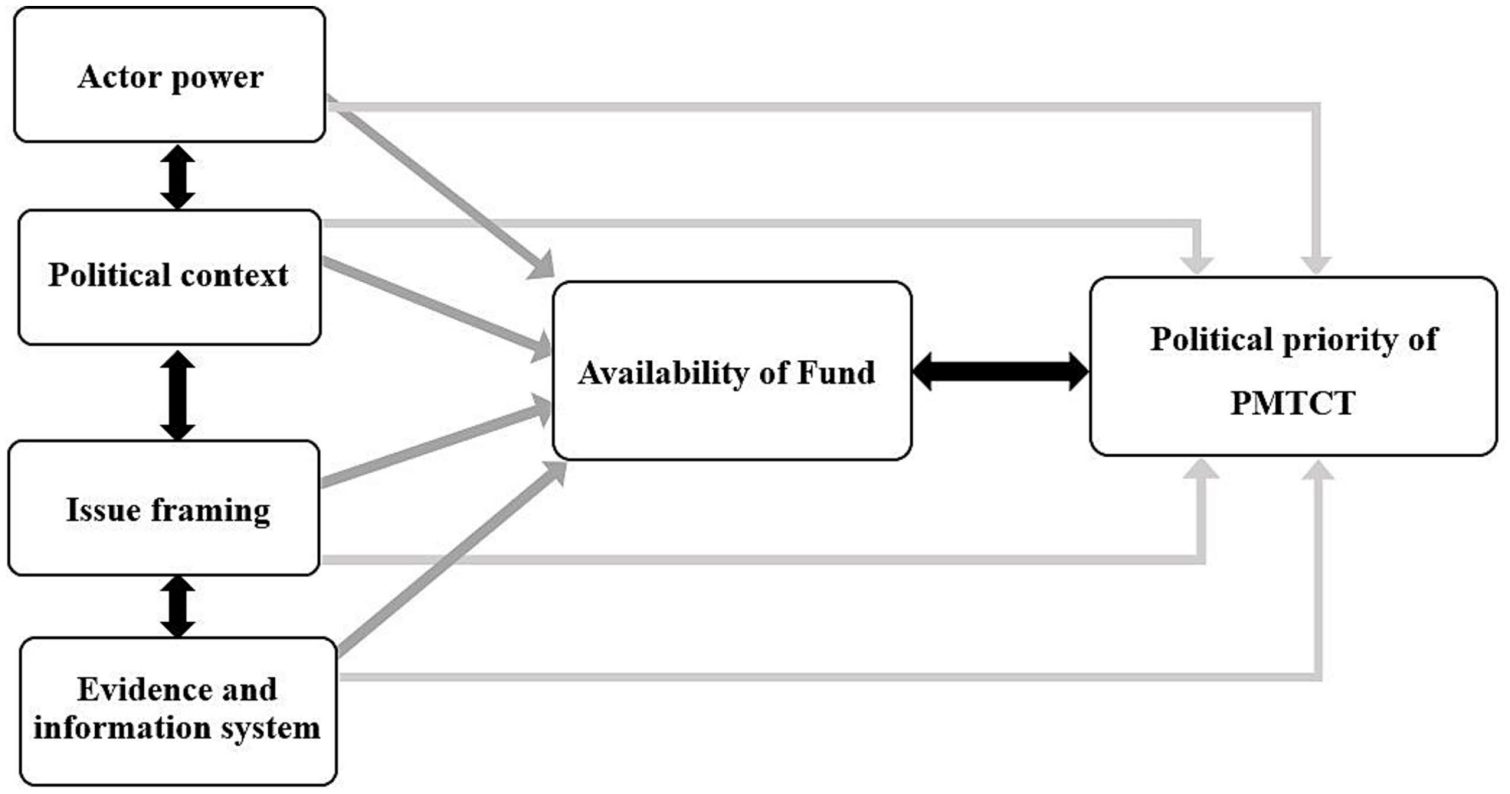
The interconnected factors influencing the political prioritization of PMTCT.

Figure 1 illustrates the dynamic interaction among key determinants shaping the political prioritization of PMTCT. Actor power, political context, issue framing, and evidence systems interact in mutually reinforcing ways to influence both resource mobilization and political commitment. Strong actor networks and effective framing increase the visibility and legitimacy of PMTCT, which in turn attract domestic and international resources. Adequate resources then strengthen health information systems and institutional capacity, creating feedback that supports continued advocacy and political engagement. Conversely, weak data systems or fragmented leadership reduce visibility, discourage donor confidence, and limit resource allocation—further weakening actor influence and political momentum. The figure highlights this cyclical relationship, showing that sustained prioritization emerges only when all domains function synergistically within a supportive policy environment.

### 4.3 Implications

This study highlights that sustained political priority for PMTCT depends on strategic advocacy that aligns actors, ideas, and resources. For those working to elevate PMTCT on national and global health agendas, several key actions are recommended:

1. Build and strengthen advocacy coalitions. Foster multi-sectoral partnerships that link government agencies, civil society, and development partners to ensure coordinated advocacy efforts and shared objectives.
2. Reframe PMTCT for greater political appeal. Present PMTCT as both a human rights and maternal health issue to align it with national development priorities and global equity agendas.
3. Leverage global policy opportunities. Utilize international commitments—such as the SDGs and WHO’s EMTCT targets—as entry points to mobilize domestic political will and strengthen international collaboration.
4. Use evidence strategically. Employ credible data and measurable indicators to demonstrate PMTCT progress, sustain donor confidence, and reinforce transparency and accountability.
5. Advocate for sustainable financing. Promote domestic resource mobilization and foster long-term partnerships with global funders to secure predictable and equitable financial support.

### 4.4 Strengths and limitations of the study

This study offers a comparative policy analysis that reveals insights into the determinants of PMTCT prioritization across three diverse country contexts. Such insights are also valuable for addressing other neglected health issues in LMICs. The use of the Shiffman and Smith framework to engage with the complex political environments of these African countries enabled clear, distinctive evidence-based outcomes. It helped fully understand how PMTCT prioritization is shaped and sustained across contexts. It offers valuable insights for analyzing health policy at both national and global levels; however, it places greater emphasis on the global agenda. Using inductive reasoning, this study adapted the framework to better capture context-specific national factors. Among these, resource provision emerged as a key determinant of political prioritization for PMTCT. This finding suggests the framework can be refined to accurately reflect how national resource allocation influences policy agendas.

Our study is limited by its reliance on secondary data, which may miss recent policy changes and stakeholder views. Future research should focus on primary data collection via interviews with policymakers, donors, and healthcare providers to enhance understanding of national decision-making processes. Additionally, the use of policy and program documents may result in aspirational and donor-driven narratives bias, as official texts frequently highlight stated objectives and achievements while underreporting implementation obstacles, and may overstate donor influence in aid-dependent contexts (130, 131). In this study, we address this by triangulating government policies, donor reports, and peer-reviewed literature, and interpreting political commitment through observable policy indicators—such as funding trends, service expansion, and monitoring systems—instead of relying solely on stated intentions.

Another limitation is the low data availability in some themes, which constrained the full triangulation. Also, limited secondary data on syphilis compared to HIV in the three countries hampered understanding the differences between the priorities of the two diseases. In Sudan, the recent consideration of PMTCT of syphilis in the national agenda complicates access to written data. However, this scarcity highlights the neglect of PMTCT of syphilis in Sudan. By grounding our analysis in cross-case comparison and applying a widely accepted framework that applies identical criteria across multiple settings, we mitigate the impact of data gaps that occur in some isolated cases.

## 5 Conclusion

This comparative analysis demonstrates that political prioritization of PMTCT emerges from the dynamic interaction of multiple determinants—actor networks, policy framing, political context, data systems, and resource provision—rather than from any single factor. Ghana and Mozambique achieved stronger national commitment by aligning advocacy coalitions, credible evidence, and financing within supportive political environments, while Sudan’s limited progress reflected weak coordination and fragmented engagement. Sustained prioritization, therefore, requires an integrated approach that simultaneously strengthens these interlinked domains. Strategic advocacy, use of reliable data, and stable resource mobilization are essential to maintain political attention, translating global EMTCT goals into durable national action to eliminate MTCT of HIV and syphilis.

## Data Availability

The original contributions presented in the study are included in the article/Supplementary material, further inquiries can be directed to the corresponding authors.

## Glossary

ANC: Antenatal Care
ART: Antiretroviral Therapy
DREAM: Drug Resource Enhancement against AIDS and Malnutrition Program
EMTCT: Elimination of Mother-to-Child Transmission
HIS: Health Information Systems
LMICs: Low- and Middle-Income Countries
MDGs: Millennium Development Goals
MOH: Ministry of Health
MTCT: Mother-to-Child Transmission
NGO: Non-Governmental Organization.
OAFLAD: Organization of African First Ladies for Development
PEPFAR: President’s Emergency Plan for AIDS Relief
PMTCT: Prevention of Mother-to-Child Transmission
SDGs: Sustainable Development Goals
TAP: Treatment Acceleration Project
UNAIDS: Joint United Nations Program on HIV/AIDS
UNGASS: United Nations General Assembly Special Sessions
UNICEF: United Nations International Children’s Emergency Fund
USAID: United States Agency for International Development
WHO: World Health Organization
