# Supplementary material for: Political prioritization of prevention of mother-to-child transmission of HIV and syphilis: a qualitative comparative policy analysis in Ghana, Mozambique, and Sudan
Source: Front Public Health. 2026 May 11;14:1751253. doi: 10.3389/fpubh.2026.1751253 (PMC13199310; doi:10.3389/fpubh.2026.1751253)
Supplement: Supplementary file 1 [file Data_Sheet_1.pdf]

Supplementary Material

Policy Documents and Data Sources

1. Governmental Documents

1.1. Ghana

- Ghana AIDS Commission (GAC). Ghana's Progress Report on The United Nations General Assembly Special Session (UNGASS) Declaration of Commitment on HIV And AIDS (January 2008 –December 2009). Ghana: Ghana AIDS Control program, 2010.
- National AIDS Control Council (NACP). Annual Report 2019. Ghana: NACP, 2020.
- Ghana AIDS Commission (GAC). Country AIDS Response Progress Report - Ghana (January 2013 – December 2014). Ghana: GAC, 2015.
- Ghana AIDS Commission (GAC). Annual Report 2020. Ghana: GAC, 2021.
- Ghana AIDS Commission (GAC). National HIV/AIDS AND STI Policy. Ghana: GAC, 2004.
- Ghana AIDS Commission (GAC). National HIV/AIDS strategic Framework II 2006-2010. Ghana: GAC, 2005.
- Ghana AIDS Commission (GAC). National HIV and AIDS Strategic Plans (2016-2020). Ghana: GAC, 2016.
- Ghana AIDS Commission (GAC). National HIV and AIDS Strategic Plans (2021- 2025). Ghana: GAC, 2020.
- National AIDS Control Council (NACP). Annual Report 2020. Ghana: NACP, 2021.

1.2. Mozambique

- National AIDS Control Council (CNCS). The Global AIDS Response Progress Report (GARPR), 2010 - 2011. Mozambique: CNCS, Joint United Nations Programme on HIV and AIDS (UNAIDS).
- Joint United Nations Programme on HIV/AIDS (UNAIDS). First Lady of Mozambique appointed as Patron of the Global Plan: UNAIDS; 2013 [cited 2023 February 13]. Available from: https://www.unaids.org/en/resources/presscentre/featurestories/2013/july/20130731mozambique.
- Ministry of Health. Annual Report 2020, Annual Report on HIV/AIDS-Related Activities [in Portuguese]. Mozambique: Ministry of Health, 2021.
- National AIDS Control Council (CNCS). Global AIDS Response Progress Report. Mozambique: CNCS, 2016.
- National AIDS Control Council (CNCS). United Nations General Assembly Special Session on HIV/AIDS, Progress Report 2008 - 2009. Mozambique: CNCS, 2010.

1.3. Sudan

- Sudan National AIDS Control Program (SNAP). Global AIDS Response Progress Reporting 2010 – 2011. Sudan: SNAP, 2012. https://www.unaids.org/sites/default/files/country/documents/ce_SD_Narrative_Report[1].pdf
- Sudan National AIDS Control Program (SNAP). Standard Operating Procedures for Health Care Providers in Maternatity Hospitals that Offer PMTCT Services in Sudan. Sudan: SNAP, 2015.
- Sudan National AIDS Control Program (SNAP). Global AIDS Response Progress Reporting 2012 – 2013. Sudan: SNAP, 2014.

https://www.unaids.org/sites/default/files/country/documents/SDN_narrative_report_2014.pdf

- Sudan National AIDS Control Program (SNAP). Country progress report- Sudan. Sudan: SNAP, 2020. https://www.unaids.org/sites/default/files/country/documents/SDN_2020_countryreport.pdf
- Sudan National AIDS Control Program (SNAP). National HIV and AIDS Strategic Plan (2010-2014). Sudan: SNAP, Sudan Federal Ministry of Health, 2010.
- United Nations Country Team, Government of Sudan. Sudan Millennium Development Goals Interim Unified Report Programme UND; 2004.
- Mahmoud AE. Sentinel Serosurveillance Survey among Pregnant Women Attending ANC Clinics in Sudan – Round (6), 2019 ANC HIV Sentinel Sero-survey Technical Report. Sudan: Sudan National AIDS Control Program, Federal Ministry of Health, 2020.

2. Non-Governmental Organization

- World Health Organization (WHO). Strengthening district health sector responses to HIV/AIDS in Sub-Saharan Africa: an evaluation of the WHO/OPEC Fund Multi-country Initiative on HIV/AIDS. Geneva: WHO, 2006.
- Organization of African First Ladies for Development (OAFLA). Adolescents and youth-friendly health services_ Harnessing the demographic dividend through investment in youth. OAFLA, 2017.
- Joint United Nations Programme on HIV/AIDS (UNAIDS). African First Ladies advocate for the ending AIDS epidemic among young women and adolescent girls: UNAIDS; 2015 [cited 2023 February 14]. Available from: https://www.unaids.org/en/resources/presscentre/featurestories/2015/june/20150616_OAFLA
- Mothers2mothers. Our work in Ghana: Mothers2mothers; 2021 [cited 2023 January 6]. Available from: https://m2m.org/what-we-do/where-we-work/ghana/.
- Greenblott K. The Partnership for HIV-Free Survival (PHFS). Maputo, Mozambique: FHI 360's Food and Nutrition Technical Assistance III Project (FANTA), 2013.
- Mothers2mothers. Mothers2mothers Launches in Mozambique: Mothers2mothers; 2018 [cited 2023 January 17]. Available from: https://m2m.org/2018/01/31/mothers2mothers-launches-mozambique/.
- Fraser N, Benedikt C, Obst M, Masaki E, Görgens M, Stuart R, et al. Sudan's HIV response: value for money in a low-level HIV epidemic. Washington, D.C: The World Bank, 2014.
- World Health Organization (WHO). Strategic framework for the elimination of new HIV infections among children in Africa by 2015. Geneva: WHO, 2013.
- U.S. President's Emergency Plan for AIDS Relief (PEPFAR). Country Operational Plan (COP 2022): Strategic Direction Summary. Mozambique: PEPFAR-Mozambique, 2022.
- World Health Organization (WHO). Investment case for eliminating mother-to-child transmission of syphilis: promoting better maternal and child health and stronger health systems. Geneva: WHO, 2012.
- World Health Organization (WHO). Global guidance on criteria and processes for validation: elimination of mother-to-child transmission of HIV and syphilis. Geneva: WHO, 2014.
- World Health Organization (WHO). Global guidance on criteria and processes for validation: Elimination of Mother-to-Child Transmission of HIV and Syphilis, 2nd edition. Geneva: WHO, 2017.
- Joint United Nations Programme on HIV/AIDS (UNAIDS). Global Plan towards the

Elimination of New HIV Infections among Children by 2015 and Keeping their Mothers Alive. Geneva 27, Switzerland: UNAIDS; 2011.

- World Health Organization (WHO). Regional Office for the Eastern Mediterranean, The situation of HIV testing and counselling policies and practices in the Eastern Mediterranean Region. Egypt: WHO, 2011.
- World Health Organization (WHO). Treatment Acceleration Project (TAP): Completion report of TAP 2005-2008. Geneva WHO, 2009.
- U.S. President’s Emergency Plan for AIDS Relief (PEPFAR). Mozambique Sustainability Index and Dashboard. Mozambique: PEPFAR, 2019.
- World Health Organization (WHO), the Eastern Mediterranean Region (EMRO). Assessment of Sudan’s health information system 2020. Cairo: Regional Office for the Eastern Mediterranean, WHO, 2022.
- Joint United Nations Programme on HIV/AIDS (UNAIDS). Start Free Stay Free AIDS Free. Geneva: UNAIDS, 2019.
- Family Health International (FHI). Mozambique Final Report October 2002–September 2006 for USAID’s Implementing AIDS Prevention and Care (IMPACT) Project. Arlington, USA: FHI, 2007.
- United Nations Children’s Fund (UNICEF). Countdown To Zero, Elimination of New HIV Infections Among Children by 2015 And Keeping Their Mothers Alive, Mozambique: UNICEF, 2013.
- U.S. President’s Emergency Plan for AIDS Relief (PEPFAR). Mozambique Country Operational Plan, COP 2020. Mozambique PEPFAR, 2020.
- World Health Organization (WHO). Progress Report 2006: Regional HIV/AIDS Treatment Acceleration Project (TAP) in Burkina Faso, Ghana and Mozambique. Geneva: WHO, 2007.

3. Literature

- Dassah ET, Adu-Sarkodie Y, Mayaud P. Rollout of rapid point of care tests for antenatal syphilis screening in Ghana: healthcare provider perspectives and experiences. BMC Health Serv Res. 2018;18(1):130. Epub 20180220. doi: 10.1186/s12913-018-2935-y. PubMed PMID: 29458363; PubMed Central PMCID: PMCPMC5819248.
- Stover KE, Shrestha R, Tsambe I, Mathe PP. Community-Based Improvements to Increase Identification of Pregnant Women and Promote Linkages to Antenatal and HIV Care in Mozambique. J Int Assoc Provid AIDS Care. 2019;18:1-8. doi: 10.1177/2325958219855623. PubMed PMID: 31232152; PubMed Central PMCID: PMCPMC6748535.
- Smith SL, Shiffman J. Setting the global health agenda: The influence of advocates and ideas on political priority for maternal and newborn survival. Soc Sci Med. 2016;166:86-93. Epub 20160809. doi: 10.1016/j.socscimed.2016.08.013. PubMed PMID: 27543685; PubMed Central PMCID: PMCPMC5034850.
- Liotta G, Mancinelli S, Nielsen-Saines K, Gennaro E, Scarcella P, Magid NA, et al. Reduction of maternal mortality with highly active antiretroviral therapy in a large cohort of HIV-infected pregnant women in Malawi and Mozambique. PLoS One. 2013;8(8):e71653. Epub 20130819. doi: 10.1371/journal.pone.0071653. PubMed PMID: 23990966; PubMed Central PMCID: PMCPMC3747183.
- Kiragu K, Collins L, Von Zinkernagel D, Mushavi A. Integrating PMTCT Into Maternal, Newborn, and Child Health and Related Services: Experiences From the Global Plan Priority Countries. J Acquir Immune Defic Syndr. 2017;75 Suppl 1:S36-s42. doi: 10.1097/qai.0000000000001323. PubMed PMID: 28398995.
- Banze AR, Homo BP, Mussá TN, Baltazar CS, Boothe MA. Evaluation of prevention of mother-to-child transmission national health information system for HIV/AIDS, in southern

region of Mozambique, April to November 2016. Pan Afr Med J. 2021;38:26. Epub 20210112. doi: 10.11604/pamj.2021.38.26.24255. PubMed PMID: 33777294; PubMed Central PMCID: PMCPMC7955592.

- Idris AK. Factors influencing access and utilization of prevention of mother to child transmission (PMTCT) of HIV services in Sudan [Thesis]: Amsterdam, The Netherlands: Free University of Amsterdam; 2012.
- Badawi MM, Atif MS, Mustafa YY. Systematic review and meta-analysis of HIV, HBV and HCV infection prevalence in Sudan. Virol J. 2018;15(1):148. Epub 20180925. doi: 10.1186/s12985-018-1060-1. PubMed PMID: 30253805; PubMed Central PMCID: PMCPMC6157049.
- Abdelmola AO. Prevalence and factors associated with syphilis among pregnant women attending antenatal care, Khartoum State, Sudan. Int J Adv Med. 2018;5(2):2-6.
- Nagi AM, Allah HAW, Khalil OM. Seroprevalence of syphilis among pregnant women in the Tri-capital, Khartoum, Sudan. Res J Med Sc. 2008;3:48-52.
- Dassah E, Opoku BK, Adu-Sarkodie Y, Louie K, Mayaud P, Mabey D. Screening for syphilis during pregnancy in Ghana: the role of new rapid point-of-care diagnostic tests. Ghana: Programme for Research and Capacity Building in Sexual and Reproductive Health and HIV in Developing Countries, 2010.
- Liljestrand J, Bergström S, Nieuwenhuis F, Hederstedt B. Syphilis in pregnant women in Mozambique. Genitourin Med. 1985;61(6):355-8. doi: 10.1136/sti.61.6.355. PubMed PMID: 3910543; PubMed Central PMCID: PMCPMC1011858.
- Bique Osman N, Challis K, Folgosa E, Cotiro M, Bergström S. An intervention study to reduce adverse pregnancy outcomes as a result of syphilis in Mozambique. Sex Transm Infect. 2000;76(3):203-7. doi: 10.1136/sti.76.3.203. PubMed PMID: 10961199; PubMed Central PMCID: PMCPMC1744159.
- VanDeusen A, Paintsil E, Agyarko-Poku T, Long EF. Cost effectiveness of option B plus for prevention of mother-to-child transmission of HIV in resource-limited countries: evidence from Kumasi, Ghana. BMC Infect Dis. 2015;15:130. Epub 20150318. doi: 10.1186/s12879-015-0859-2. PubMed PMID: 25887574; PubMed Central PMCID: PMCPMC4374181.
- Levin CE, Steele M, Atherly D, García SG, Tinajeros F, Revollo R, et al. Analysis of the operational costs of using rapid syphilis tests for the detection of maternal syphilis in Bolivia and Mozambique. Sex Transm Dis. 2007;34(7 Suppl):S47-54. doi: 10.1097/01.olq.0000245986.62775.b6. PubMed PMID: 17220812.
